# Pharmaco-Metabolomics of Inhaled Corticosteroid Response in Individuals with Asthma

**DOI:** 10.3390/jpm11111148

**Published:** 2021-11-04

**Authors:** Priyadarshini Kachroo, Joanne E. Sordillo, Sharon M. Lutz, Scott T. Weiss, Rachel S. Kelly, Michael J. McGeachie, Ann Chen Wu, Jessica A. Lasky-Su

**Affiliations:** 1 Department of Medicine, Channing Division of Network Medicine, Brigham and Women’s Hospital and Harvard Medical School, Boston, MA 02115, USA; reprk@channing.harvard.edu (P.K.); scott.weiss@channing.harvard.edu (S.T.W.); hprke@channing.harvard.edu (R.S.K.); remmg@channing.harvard.edu (M.J.M.); 2 PRecisiOn Medicine Translational Research (PROMoTeR) Center, Department of Population Medicine, Harvard Medical School and Harvard Pilgrim Health Care, Boston, MA 02215, USA; rejoa@channing.harvard.edu (J.E.S.); sharon.m.lutz@gmail.com (S.M.L.); ann.wu@childrens.harvard.edu (A.C.W.)

**Keywords:** asthma, inhaled corticosteroids, metabolites, metabolomics, age interactions

## Abstract

Metabolomic indicators of asthma treatment responses have yet to be identified. In this study, we aimed to uncover plasma metabolomic profiles associated with asthma exacerbations while on inhaled corticosteroid (ICS) treatment. We determined whether these profiles change with age from adolescence to adulthood. We utilized data from 170 individuals with asthma on ICS from the Mass General Brigham Biobank to identify plasma metabolites associated with asthma exacerbations while on ICS and examined potential effect modification of metabolite-exacerbation associations by age. We used liquid chromatography–high-resolution mass spectrometry-based metabolomic profiling. Sex-stratified analyses were also performed for the significant associations. The age range of the participating individuals was 13–43 years with a mean age of 33.5 years. Of the 783 endogenous metabolites tested, eight demonstrated significant associations with exacerbation after correction for multiple comparisons and adjusting for potential confounders (Bonferroni *p* value < 6.2 × 10^−4^). Potential effect modification by sex was detected for fatty acid metabolites, with males showing a greater reduction in their metabolite levels with ICS exacerbation. Thirty-eight metabolites showed suggestive interactions with age on exacerbation (nominal *p*-value < 0.05). Our findings demonstrate that plasma metabolomic profiles differ for individuals who experience asthma exacerbations while on ICS. The differentiating metabolites may serve as biomarkers of ICS response and may highlight metabolic pathways underlying ICS response variability.

## 1. Introduction

Asthma imparts a tremendous global health and economic burden, affecting over 350 million people worldwide [1,2,3,4]. While several genetic variants have been determined to influence an individual’s asthma susceptibility [5,6,7,8], asthma also has substantial environmental triggers [9] and the majority of cases arise from complex interactions between both factors. Inhaled corticosteroids (ICS) are the most commonly used controller medications for the treatment of individuals with moderate to severe asthma [10]. However, approximately 25 to 35% of asthma patients either do not respond or respond poorly to ICS [11,12]. Early identification of patients as responders or non-responders to ICS therapy will enhance treatment efficacy and will minimize the overall impact of ICS side effects by avoiding treatment in individuals who are non-responders [13,14,15,16,17,18].

Metabolomics is one type of high-dimensional “omics” data that can be leveraged to identify biomarkers of medication response [19,20]. Metabolomics, the systematic analysis of small molecules in a biological sample, provides an integrated profile of genetics, environmental exposures, and phenotype, reflecting the “net results” of genetic, transcriptomic, proteomic, and environmental interactions, making it ideally suited to the study of asthma etiology and phenotypes [21,22]. Pharmacometabolomics is an emerging discipline that has the potential to improve our understanding of the mechanistic effects of drugs and inform precision medicine initiatives for individuals with asthma on ICS [23].

Using data from our Age-Dependent Pharmacogenomics of Asthma Treatment (ADAPT) study, [24] we examined plasma metabolomics to identify metabolites associated with asthma exacerbations while on ICS. In secondary analyses, we tested metabolite interactions with age to determine if pharmacometabolomic predictors of ICS response were age-dependent. We leveraged Mass General Brigham Biobank (MGBB) and electronic medical health record (EMR) data to conduct our analyses.

## 2. Methods

### 2.1. Study Population: Mass General Brigham Biobank

The Mass General Brigham Biobank (MGBB) (https://biobank.partners.org, access date 30 June 2020) is a collection of DNA, serum, and plasma samples from 81,502 fully consented subjects, linked to the Research Patient Data Registry (RPDR), a data warehouse that gathers data from multiple electronic medical health record (EMR) systems and stores it in an SQL Server database. We applied a validated phenotyping algorithm [25] for asthma diagnosis (positive predictive value > 85%) using the RPDR and identified asthma cases with the most recent diagnosis after the plasma collection date. Using the EMR data, we obtained information on asthma medication use for the identified asthma cases. We created a binary measure of ICS use using information on the total number of ICS prescriptions for the following medications: beclomethasone dipropionate, budesonide, ciclesonide, inhaled dexamethasone, flunisolide, fluticasone, fluticasone/salmeterol, mometasone, and triamcinolone. Among the asthma cases with ICS intake, a quantitative measure of exacerbation was defined using episodes of exacerbation based on the physician’s report. Subjects were classified as “with exacerbation” if they either had at least one episode in the reported count/frequency of the number of exacerbations or a yes to any of the following criteria: (1) mild intermittent asthma with (acute) exacerbation, (2) mild persistent asthma with (acute) exacerbation, (3) moderate persistent asthma with (acute) exacerbation, (4) severe persistent asthma with (acute) exacerbation, and (5) unspecified asthma with (acute) exacerbation. Otherwise, they were classified as “without exacerbation”. This study was approved by the IRB of Mass General Brigham and all study participants provided written consent at enrollment.

### 2.2. Metabolomic Profiling for Mass General Brigham Biobank

Metabolomic profiling was conducted by Metabolon Inc. (Durham, NC, USA) using four non-targeted liquid chromatography couple mass spectroscopy (LCMS) platforms, enabling the broadest coverage of the metabolome. The methods have been described in detail previously [26]. In short, four non-targeted liquid chromatography couple mass spectroscopy (LCMS) platforms were performed as follows: (1) UPLC-MS/MS under positive ionization; (2) UPLC-MS/MS under negative ionization; (3) UPLC-MS/MS, polar platform (negative ionization); and (4) GC-MS. Metabolites were identified by their mass-to-charge ratio (*m*/*z*), retention time (rt), and through a comparison to a library of purified known standards. Peaks were quantified using area-under-the-curve. Metabolite measures were median normalized across run days (with medians set to 1).

Plasma samples for the cohort were collected between October 2010 and March 2017 and were stored immediately (within 4 h) in a freezer at −80 degrees. Non-fasting plasma samples were available from all the participants, which were used for metabolomic profiling. The most recent diagnosis of asthma was chosen based on the plasma collection date. Metabolomic data were available for 171 asthmatics with ICS use in MGBB. One outlier subject with 82 exacerbations was removed from the analysis. There were 90 subjects without exacerbation (zero counts of exacerbation) and 80 subjects with at least one episode of exacerbation (Figure S1). Metabolite intensities were log transformed and pareto scaled, and missing metabolite values were imputed by replacement with half the minimum value for each metabolite in all samples. Metabolites with an interquartile range (IQR) of zero as well as those with less variance (an IQR less than 0.25) were excluded from further analysis (*n* = 143). Further, for this analysis we only considered endogenous metabolites.

### 2.3. Statistical Analysis

#### Metabolite Associations with Exacerbation and Their Effect Modification by Age

To account for the over-dispersion in the exacerbation count data, a quasi-Poisson regression was used to test the association between plasma metabolites (predictor) and the exacerbation counts (outcome). Models were adjusted for age, sex, race (White, African American, and Other), body mass index (BMI), smoking status, and time gap (in years) between the date of the first blood draw and the date until which the samples were followed for the data freeze as the data in the biobank is dynamic. The data was downloaded from the Biobank on 30 June 2020. We also explored these associations stratified by sex and further evaluated these models for age–metabolite interactions, including the interaction term (age*metabolite). The age–metabolite interactions were explored in these subjects to understand the influence of age on these metabolite associations over adulthood. Age was mean centered to reduce collinearity with the interaction term. To account for multiple testing comparisons while taking into consideration the high correlation between metabolites that exist within interconnected pathways, we applied the “effective number of independent tests” (ENT) approach [27,28,29,30,31,32], exploring a threshold of ENT85% (accounting for 85% of the total variance in metabolites; the corresponding Bonferroni *p*-value threshold was 6.2 × 10^−4^). All analyses are conducted in R version 4.0.3 [33].

## 3. Results

Clinical characteristics of participating subjects (*n* = 170) from the MGBB-cohort are summarized in Table 1. The mean age in these subjects was 33.5 years with an age range of 13–43 years. There was no significant difference between ICS-asthmatics with and without exacerbation based on age, BMI, and smoking status; however, exacerbation status differed by sex (*p* = 0.03) and race (*p* = 0.03).

### Metabolite Associations with Exacerbation and Their Effect Modification by Age and Sex

**Figure 1:**
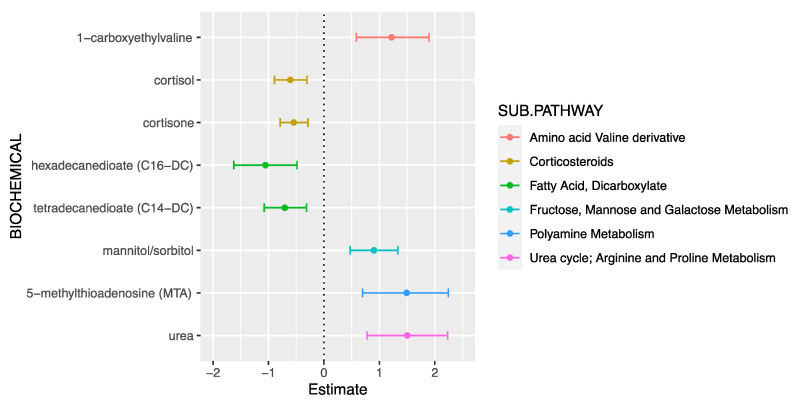
Metabolite (predictor) associations with exacerbation (outcome) in asthma cases with inhaled corticosteroid (ICS) intake at an “effective number of tests” (ENT) Bonferroni threshold *p*-value of 6.2 × 10^−4^. The figure shows the effect/β-estimates with confidence intervals on the *x*-axis and metabolites on the *y*-axis colored by their sub-pathways in the legend key.

In total, 783 endogenous metabolites remained for the downstream analyses after quality control; 482 (61.6%) of those were annotated to metabolite super pathways. Most of these annotated metabolites were lipids (38.0%) and amino acids (36.7%). There were 65 metabolites that were associated with exacerbation at a *p*-value threshold of 0.05 (Table S1). Eight of those 65 metabolites were also significant at an ENT85% (effective number of independent tests accounting for 85% of the total variance in metabolites, Table 2, Figure 1). There were four metabolites belonging to the lipid super-pathway and all demonstrated a reduction in metabolite levels with an increase in exacerbation: cortisone (β = −0.55; 95%CI =−0.79, −0.29; *p*-value = 2.90 *×* 10^−5^), cortisol (β=−0.61; 95%CI=−0.89, −0.30; *p*-value = 7.11 *×* 10^−5^), tetradecanedioate (β = −0.71; 95%CI = −1.08, −0.31; *p*-value = 3.6 *×* 10^−4^), and hexadecanedioate (β = −1.05, 95%CI = −1.62, −0.49; *p*-value = 3.7 *×* 10^−4^). The remaining four demonstrated positive associations with exacerbation and belonged to carbohydrate and amino acid super-pathways: mannitol/sorbitol (β = 0.90; 95%CI = 0.47, 1.33; *p*-value = 5.93 *×* 10^−5^), urea (β = 1.50; 95%CI = 0.78, 2.23; *p*-value = 7.78 *×* 10^−5^), 5-methylthioadenosine (β = 1.49; 95%CI = 0.70, 2.24; *p*-value = 2.2 *×* 10^−4^), and 1-carboxyethylvaline (β = 1.22, 95%CI = 0.58, 1.89; *p*-value = 3.8 *×* 10^−4^). Given known sex differences in asthma cases, we further stratified these significant associations by sex (Tables S2 and S3). All eight metabolite associations remained significant, and their direction of effect remained consistent in females. Males had a limited sample size; therefore, only the metabolites associated with lipid metabolism belonging to the corticosteroid (cortisol and cortisone) and fatty acid (hexadecanedioate and tetradecanedioate) sub-pathways retained significance in males who also demonstrated increased reduction in these metabolite levels.

Of the 783 tested metabolites, 38 metabolites demonstrated a significant interaction with age in association with exacerbation at a nominal *p*-value threshold of 0.05; 10 of those were significant even at a more stringent *p*-value threshold of 0.01 (Table S4), including the named metabolites: lactate, fructose, glycoursodeoxycholate, 3-hydroxydecanoate, ursodeoxycholate, and phenylalanyltryptophan. This suggests that these metabolites may be modified over age in asthma cases with exacerbation and could potentially be a target for age-related interventions. We did not identify a significant age–metabolite interaction effect for the eight metabolites as highlighted above. 

## 4. Discussion

Pharmacometabolomics is an emerging approach with the potential to identify biomarkers of treatment response as well as the metabolic pathways that underlie drug response variability [34]. Pharmacometabolomic indicators of treatment responses may ultimately help reduce asthma morbidity by increasing the precision of asthma treatment regimens. In this work, we identified plasma metabolomic indicators of asthma exacerbations while on ICS treatment.

We detected metabolite indicators of exacerbation while on ICS treatment from lipid and amino acid biochemical classes. Two of the top metabolites, hexadecanedioate and tetradecanedioate, are derived from omega fatty acid oxidation, a subsidiary pathway of beta-oxidation [35]. Fatty acid oxidation may have a key role in asthma pathogenesis. Emerging evidence from murine models of asthma shows that allergic inflammation in the airways increases with fatty acid oxidation enzyme activity in immune cells [36]. Furthermore, in vivo and in vitro metabolomics studies of bronchial smooth muscle cells from participants with asthma have identified beta-oxidation of fatty acids as a predictor of cell proliferation, a marker of airway remodeling [37]. Lastly, omega-fatty acid oxidation has a key role in leukotriene pathways [35]. While existing evidence shows links between fatty acid oxidation and asthma pathogenesis, ours is the first report linking fatty acid oxidation to ICS response in asthma. Our findings suggest that this metabolic pathway could be a useful target for enhancing treatment outcomes. Additional population studies, as well as functional validation models, will be necessary to fully understand the connection between fatty acid oxidation and ICS treatment response.

Our findings also show a potential link between ICS response in asthma and metabolites in amino acid metabolism pathways. Valine metabolites have previously been indicated as biomarkers of asthma case status in a study of the sputum metabolome [38]. Biomarkers of the urea cycle/arginine metabolism are also associated with lung eosinophilia and airway hyper-responsiveness in experimental murine models of asthma [39]. Arginine metabolism, and its connection to the urea cycle, has been linked to asthma case status, airflow obstruction, and severity [40,41] in epidemiology studies, and now shows a connection to ICS response in our work presented here. Additional studies in human populations as well as functional validation studies will be required to fully elucidate any connection between urea cycle metabolites and ICS response.

Lastly, we identified reduced cortisol and cortisone as metabolomic correlates of exacerbations while on ICS. Both metabolites are known markers of ICS treatment. The fact that they are negatively associated with exacerbations suggests that individuals who consistently used their ICS medication (and despite this use had higher levels of circulating cortisone and cortisol) also had less frequent exacerbations.

Two previous studies have focused on metabolomic profiling of corticosteroid-resistant asthma, both of which were conducted in pediatric populations. Fitzpatrick et al. [42] reported that sphingolipid metabolism, oxidative stress pathways (glutathione), and amino acid metabolites (involving glycine, serine, and threonine metabolism) were altered in plasma samples from children with corticosteroid refractory severe asthma. Park et al. [43] found that metabolites from tyrosine, glutathione, and catecholamine pathways differed in urine samples from patients with corticosteroid-resistant asthma. Our study of exacerbations while on ICS was conducted in participants across a wide age range (from early adolescence to mid-life). The metabolite predictors in our study were distinct from those identified in pediatric populations. There could be several explanations for why our findings differ, including different tissue types, metabolomic platforms, and life stages, which are key factors that may account for differences in the results. Given the wide age range in our biobank population, we were able to interrogate potential age by metabolite interactions, to determine if important metabolite predictors of ICS response vary by age. Metabolites associated with tryptophan (phenylalanyltryptophan), glycolysis (lactate), fructose metabolism, and bile acid metabolism (ursodeoxycholate and glycoursodeoxycholate) were the top biomarkers demonstrating a potential interaction with age. Previous studies have shown that lactate is a biomarker of exacerbations, and that serum lactase is upregulated during acute asthma treatment [44,45,46], hinting towards early onset or susceptibility to lung disease. Bile acid metabolites are associated with potential links between the gut microbiome and asthma phenotypes [47] and may also help explain the links to obesity/asthma [48]. Accounting for age by metabolite interaction in our models still did not recapitulate any of the metabolite findings from the Fitzpatrick et al. or Park et al. studies, perhaps because the lower end of the age range in our study (adolescence), did not overlap enough with the age range (mid-childhood to early adolescence) in these previous studies.

Our study showed several strengths as well as some limitations. The strengths of our study include a relatively large population (as compared to prior studies of metabolomics of ICS treatment responses), the use of untargeted metabolomics data to interrogate metabolites across multiple chemical classes, and the leverage of existing biobank samples and corresponding medical record data to answer our research question. The limitations of our study include limited power to detect age by metabolite interactions, the cross-sectional nature of metabolomic profiling and phenotype assessment, and the absence of pediatric participants (to compare with metabolomics of ICS response in adolescents and adults). In the present work we focused solely on metabolomics. However, future studies that incorporate additional “omics” data, for example transcriptomics and/or epigenomics, may uncover regulatory signals underlying associations of metabolites and phenotypes.

In conclusion, our findings demonstrate that plasma metabolomic profiles differ for individuals who experience asthma exacerbations while on ICS. These metabolites may serve as biomarkers of ICS response and may highlight metabolic pathways underlying ICS response variability.

## Figures and Tables

**Table 1:** Clinical characteristics of the subjects in the MGBB-cohort.

Clinical Characteristics	All Subjects (*n* = 170)	Exacerbation		*p*-Value *
		Presence (*n* = 80)	Absence (*n* = 90)	
Sex, *n* (%)				0.03
Female	136 (80.0)	70 (87.5)	66 (73.3)	
Male	34 (20.0)	10 (12.5)	24 (26.7)	
Race, *n* (%)				0.03
African American	23 (13.5)	15 (18.8)	8 (8.9)	
White	132 (77.6)	55 (68.8)	77 (85.6)	
Others	15 (8.8)	10 (12.5)	5 (5.6)	
Smoking, *n* (%)				0.25
No	127 (74.7)	56 (70.0)	71 (78.9)	
Yes	43 (25.3)	24 (30.0)	19 (21.1)	
Exacerbation counts, mean (SD)	2.2 (4.6)	4.6 (5.8)	0.0 (0.0)	NA
Age, mean (SD)	33.5 (7.1)	34.6 (5.9)	32.6 (7.8)	0.06
BMI kg/m^2^, mean (SD)	29.8 (8.6)	30.6 (8.6)	29.0 (8.5)	0.23

* Significance of difference was evaluated using chi-square test for categorical variables and two-sample t-test for continuous variables. Data for body mass index (BMI) was missing for five subjects. Exacerbation counts are shown for clarity since the quantitative variable was used to get the best power for models. Abbreviations: BMI, body mass index; SD, standard deviation.

**Table 2:** Metabolite (predictor) associations with exacerbation (outcome) in asthma cases with inhaled corticosteroid (ICS) intake at an “effective number of tests” (ENT) *.

Metabolite	Super-Pathway	Sub-Pathway	β (95%CI)	*p*-Value
Cortisone	Lipid	Corticosteroids	−0.55 (−0.79, −0.29)	2.90 × 10^−5^
Cortisol	Lipid	Corticosteroids	−0.61 (−0.89, −0.30)	7.11 × 10^−5^
Tetradecanedioate (C14-DC)	Lipid	Fatty acid, dicarboxylate	−0.71 (−1.08, −0.31)	3.6 × 10^−4^
Hexadecanedioate (C16-DC)	Lipid	Fatty acid, dicarboxylate	−1.05 (−1.62, −0.49)	3.7 × 10^−4^
Mannitol/Sorbitol	Carbohydrate	Fructose, mannose, and galactose metabolism	0.90 (0.47, 1.33)	5.93 × 10^−5^
Urea	Amino Acid	Urea cycle; arginine and proline metabolism	1.50 (0.78, 2.23)	7.78 × 10^−5^
5-methylthioadenosine (MTA)	Amino Acid	Polyamine metabolism	1.49 (0.70, 2.24)	2.2 × 10^−4^
1-carboxyethylvaline	Amino Acid	Valine derivative	1.22 (0.58, 1.89)	3.8 × 10^−4^

* Bonferroni threshold *p*-value of 6.2 × 10^−4^. The table is sorted by sub-pathway followed by *p*-value.

## Data Availability

Requests for raw data, analyzed data and materials for specific scientific purposes will be reviewed by the cohort and contact PIs for the studies to determine if the request is subject to intellectual property or confidentiality obligations. Data and materials presented in this study that can be shared will be released using a Material Transfer Agreement. Appropriate IRB approvals may be required to access de-identified data in particular data from electronic medical health records. Data will either be shared through an institutional data sharing agreement or arrangements will be made for analyses to be conducted remotely without the need for data transfer.

## Supplementary Materials

The following are available online at https://www.mdpi.com/article/10.3390/jpm11111148/s1, Figure S1: Distribution of counts/episodes of exacerbation for each asthma case in MGBB-cohort; Table S1. Metabolite associations with exacerbation counts (continuous outcome/number of exacerbations). The list is sorted by the *p*-value (glm quasipoisson). 65 metabolites in the metabolite as main effect model were significant at nominal P threshold. However, top 8 metabolites were also significant based on effective number of tests (ENT) bonferroni threshold Pvalue of 6.2 × 10−4; with 81 PCs explaining more than 85% of the variance in the data; Table S2. Metabolite associations with exacerbation counts in females (n = 133 females, continuous outcome/number of exacerbations). The list is sorted by the *p*-value (glm quasipoisson). We tested only eight metabolites that were significant in Table 1 at ENT85% bonferroni threshold of 6.2 × 10−4. All except hexadecanedioate would be significant if we use a bonferroni threshold of 6.25 × 10−3 (0.05/8 tested metabolites); Table S3. Metabolite associations with exacerbation counts in males (n = 32 males, continuous outcome/number of exacerbations). The list is sorted by the *p*-value (glm quasipoisson). We tested only eight metabolites that were significant in Table 1 at ENT85% bonferroni threshold of 6.2 × 10−4. We have less power here to detect robust associations); Table S4: Age-metabolite associations with exacerbation counts (continuous outcome/number of exacerbations). The list is sorted by the *p*-value of the age-metabolite analysis (glm quasipoisson). 38 metabolites were significant at nominal threshold, however none passed the ENT Pvalue threshold. The results from the metabolite and age as main effect for the 38 metabolites are also included.

## References

[1] Global Initiative for Asthma (2021). Global Strategy for Asthma Management and Prevention. www.ginasthma.org.

[2] Masoli M., Fabian D., Holt S., Beasley R. (2004). The global burden of asthma: Executive summary of the GINA Dissemination Committee report. Allergy.

[3] Becker A.B., Abrams E.M. (2017). Asthma guidelines: The Global Initiative for Asthma in relation to national guidelines. Curr. Opin. Allergy Clin. Immunol..

[4] CDC (2018). Data, Statistics, and Surveillance—Asthma Surveillance Data.

[5] Greally M., Jagoe W.S., Greally J. (1982). The genetics of asthma. Ir. Med. J..

[6] Dold S., Wjst M., von Mutius E., Reitmeir P., Stiepel E. (1992). Genetic risk for asthma, allergic rhinitis, and atopic dermatitis. Arch. Dis. Child..

[7] Jenkins M.A., Hopper J.L., Giles G.G. (1997). Regressive logistic modeling of familial aggregation for asthma in 7,394 population-based nuclear families. Genet. Epidemiol..

[8] Sharma S., Chhabra D., Kho A.T., Hayden L., Tantisira K.G., Weiss S.T. (2014). The genomic origins of asthma. Thorax.

[9] Louisias M., Ramadan A., Naja A.S., Phipatanakul W. (2019). The Effects of the Environment on Asthma Disease Activity. Immunol. Allergy Clin. N. Am..

[10] Crompton G. (2006). A brief history of inhaled asthma therapy over the last fifty years. Prim. Care Respir. J..

[11] Martin R.J., Szefler S.J., King T.S., Kraft M., Boushey H.A., Chinchilli V.M., Craig T.J., DiMango E.A., Deykin A., Fahy J.V. (2007). The Predicting Response to Inhaled Corticosteroid Efficacy (PRICE) trial. J. Allergy Clin. Immunol..

[12] Wu Y.-F., Su M.-W., Chiang B.-L., Yang Y.-H., Tsai C.-H., Lee Y.L. (2017). A simple prediction tool for inhaled corticosteroid response in asthmatic children. BMC Pulm. Med..

[13] Duplantier J.E., Nelson R.P.J., Morelli A.R., Good R.A., Kornfeld S.J. (1998). Hypothalamic-pituitary-adrenal axis suppression associated with the use of inhaled fluticasone propionate. J. Allergy Clin. Immunol..

[14] Guilbert T.W., Morgan W.J., Zeiger R., Mauger D.T., Boehmer S.J., Szefler S.J., Bacharier L.B., Lemanske R.F., Strunk R.C., Allen D.B. (2006). Long-term inhaled corticosteroids in preschool children at high risk for asthma. N. Engl. J. Med..

[15] Allen D.B. (2006). Effects of inhaled steroids on growth, bone metabolism, and adrenal function. Adv. Pediatr..

[16] Lapi F., Kezouh A., Suissa S., Ernst P. (2013). The use of inhaled corticosteroids and the risk of adrenal insufficiency. Eur. Respir. J..

[17] Keeley D. (2019). Inhaled corticosteroids for asthma: Guidance is inconsistent. BMJ.

[18] Gurnell M., Heaney L.G., Price D., Menzies-Gow A. (2021). Long-term corticosteroid use, adrenal insufficiency and the need for steroid-sparing treatment in adult severe asthma. J. Intern. Med..

[19] Wang C., Jiang S., Zhang S., Ouyang Z., Wang G., Wang F. (2021). Research Progress of Metabolomics in Asthma. Metabolites.

[20] Santos A., Pité H., Chaves-Loureiro C., Rocha S.M., Taborda-Barata L. (2021). Metabolic Phenotypes in Asthmatic Adults: Relationship with Inflammatory and Clinical Phenotypes and Prognostic Implications. Metabolites.

[21] Kaddurah-Daouk R., Kristal B.S., Weinshilboum R.M. (2008). Metabolomics: A global biochemical approach to drug response and disease. Annu. Rev. Pharmacol. Toxicol..

[22] di Palmo E., Cantarelli E., Catelli A., Ricci G., Gallucci M., Miniaci A., Pession A. (2021). The Predictive Role of Biomarkers and Genetics in Childhood Asthma Exacerbations. Int. J. Mol. Sci..

[23] Kaddurah-Daouk R., Weinshilboum R.M. (2014). Pharmacometabolomics: Implications for clinical pharmacology and systems pharmacology. Clin. Pharmacol. Ther..

[24] Sordillo J.E., McGeachie M., Lutz S.M., Lasky-Su J., Tantisira K., Tsai C.H., Dahlin A., Kelly R., Wu A.C. (2019). Longitudinal analysis of bronchodilator response in asthmatics and effect modification of age-related trends by genotype. Pediatr. Pulmonol..

[25] Yu S., Liao K.P., Shaw S.Y., Gainer V.S., E Churchill S., Szolovits P., Murphy S.N., Kohane I.S., Cai T. (2015). Toward high-throughput phenotyping: Unbiased automated feature extraction and selection from knowledge sources. J. Am. Med. Inform. Assoc..

[26] Kelly R.S., Virkud Y., Giorgio R., Celedón J.C., Weiss S.T., Lasky-Su J. (2017). Metabolomic profiling of lung function in Costa-Rican children with asthma. Biochim. Biophys. Acta Mol. Basis Dis..

[27] Li M.-X., Yeung J.M.Y., Cherny S.S., Sham P.C. (2012). Evaluating the effective numbers of independent tests and significant p-value thresholds in commercial genotyping arrays and public imputation reference datasets. Hum. Genet..

[28] Nyholt D.R. (2004). A simple correction for multiple testing for single-nucleotide polymorphisms in linkage disequilibrium with each other. Am. J. Hum. Genet..

[29] Kelly R.S., Boulin A., Laranjo N., Lee-Sarwar K., Chu S., Yadama A.P., Carey V., Litonjua A.A., Lasky-Su J., Weiss S.T. (2019). Metabolomics and Communication Skills Development in Children; Evidence from the Ages and Stages Questionnaire. Metabolites.

[30] Huang M., Kelly R.S., Kachroo P., Chu S.H., Lee-Sarwar K., Chawes B.L., Bisgaard H., Litonjua A.A., Weiss S.T., Lasky-Su J. (2020). Plasma 25-Hydroxyvitamin D Concentrations are Associated with Polyunsaturated Fatty Acid Metabolites in Young Children: Results from the Vitamin D Antenatal Asthma Reduction Trial. Metabolites.

[31] Kelly R.S., Bayne H., Spiro A., Vokonas P., Sparrow D., Weiss S.T., Schwartz J., Nassan F.L., Lee-Sarwar K., Huang M. (2020). Metabolomic signatures of lead exposure in the VA Normative Aging Study. Environ. Res..

[32] Huang M., Kelly R.S., Chu S.H., Kachroo P., Gürdeniz G., Chawes B.L., Bisgaard H., Weiss S.T., Lasky-Su J. (2021). Maternal Metabolome in Pregnancy and Childhood Asthma or Recurrent Wheeze in the Vitamin D Antenatal Asthma Reduction Trial. Metabolites.

[33] RC Team (2020). R: A Language and Environment for Statistical Computing.

[34] Beger R.D., A Schmidt M., Kaddurah-Daouk R. (2020). Current Concepts in Pharmacometabolomics, Biomarker Discovery, and Precision Medicine. Metabolites.

[35] Miura Y. (2013). The biological significance of ω-oxidation of fatty acids. Proc. Jpn. Acad. Ser. B Phys. Biol. Sci..

[36] Al-Khami A.A., Ghonim M.A., Del Valle L., Ibba S.V., Zheng L., Pyakurel K., Okpechi S.C., Garay J., Wyczechowska D., Sanchez-Pino M.D. (2017). Fuelling the mechanisms of asthma: Increased fatty acid oxidation in inflammatory immune cells may represent a novel therapeutic target. Clin. Exp. Allergy J. Br. Soc. Allergy Clin. Immunol..

[37] Esteves P., Blanc L., Celle A., Dupin I., Maurat E., Amoedo N., Cardouat G., Ousova O., Gales L., Bellvert F. (2021). Crucial role of fatty acid oxidation in asthmatic bronchial smooth muscle remodelling. Eur. Respir. J..

[38] Tian M., Chen M., Bao Y.-L., Xu C.-D., Qin Q.-Z., Zhang W.-X., He Y.-T., Shao Q. (2017). Sputum metabolomic profiling of bronchial asthma based on quadruple time-of-flight mass spectrometry. Int. J. Clin. Exp. Pathol..

[39] Quinn K.D., Schedel M., Nkrumah-Elie Y., Joetham A., Armstrong M., Cruickshank-Quinn C., Reisdorph N., Gelfand E.W. (2017). Dysregulation of metabolic pathways in a mouse model of allergic asthma. Allergy.

[40] Lara A., Khatri S.B., Wang Z., Comhair S.A.A., Xu W., Dweik R.A., Bodine M., Levison B.S., Hammel J., Bleecker E. (2008). Alterations of the arginine metabolome in asthma. Am. J. Respir. Crit. Care Med..

[41] Xu W., Comhair S.A.A., Janocha A.J., Lara A., Mavrakis L.A., Bennett C.D., Kalhan S.C., Erzurum S.C. (2017). Arginine metabolic endotypes related to asthma severity. PLoS ONE.

[42] Fitzpatrick A.M., Park Y., Brown L.A.S., Jones D.P. (2014). Children with severe asthma have unique oxidative stress-associated metabolomic profiles. J. Allergy Clin. Immunol..

[43] Park Y.H., Fitzpatrick A.M., Medriano C.A., Jones D.P. (2017). High-resolution metabolomics to identify urine biomarkers in corticosteroid-resistant asthmatic children. J. Allergy Clin. Immunol..

[44] Rodrigo G.J. (2014). Serum lactate increase during acute asthma treatment: A new piece of the puzzle. Chest.

[45] Loureiro C.C., Duarte I., Gomes J., Carrola J., Barros A.S., Gil A., Bousquet J., Todo-Bom A., Rocha S. (2014). Urinary metabolomic changes as a predictive biomarker of asthma exacerbation. J. Allergy Clin. Immunol..

[46] Quan-Jun Y., Jian-Ping Z., Jian-Hua Z., Yong-Long H., Bo X., Jing-Xian Z., Bona D., Yuan Z., Cheng G. (2017). Distinct Metabolic Profile of Inhaled Budesonide and Salbutamol in Asthmatic Children during Acute Exacerbation. Basic Clin. Pharmacol. Toxicol..

[47] Lee-Sarwar K.A., Lasky-Su J., Kelly R.S., Litonjua A.A., Weiss S.T. (2020). Gut Microbial-Derived Metabolomics of Asthma. Metabolites.

[48] Shore S.A., Cho Y. (2016). Obesity and Asthma: Microbiome-Metabolome Interactions. Am. J. Respir. Cell Mol. Biol..

