# Supplementary figures and images for: Pharmaco-Metabolomics of Inhaled Corticosteroid Response in Individuals with Asthma
Source: J Pers Med. 2021 Nov 4;11(11):1148. doi: 10.3390/jpm11111148 (PMC8622526; doi:10.3390/jpm11111148)

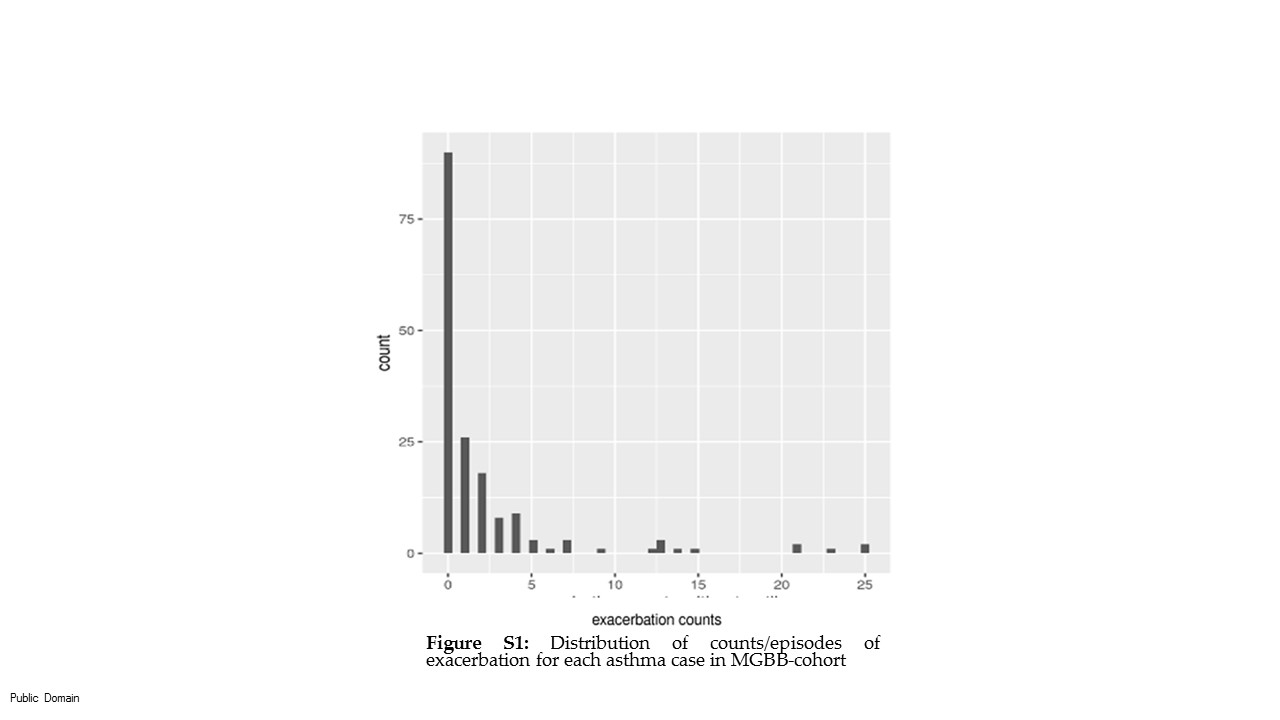

Supplement: Supplementary file 1 [file jpm-11-01148-s001.zip › Figure_S1.jpg]
